# A retrospective study of *Aeromonas hydrophila* infections at a university tertiary hospital in Saudi Arabia

**DOI:** 10.1186/s12879-023-08660-8

**Published:** 2023-10-09

**Authors:** Reham Kaki

**Affiliations:** 1 Department of Medicine, King Abdulaziz University, Jeddah, Saudi Arabia; 2 Department of Infectious Disease & Infection Control and Environmental Health, King Abdulaziz University Hospital, Jeddah, 22252 Saudi Arabia

**Keywords:** *Aeromonas hydrophila*, Comorbidity, Mortality, Nosocomial, Retrospective, Saudi Arabia, Susceptibility

## Abstract

**Background:**

*Aeromonas hydrophila* can cause a wide range of diseases and is mainly found in patients with underlying diseases. Globally the data on Aeromonas infections is limited, and no studies have been published about the situation in Saudi Arabia. The aim of this study was to investigate the risk factors, clinical presentation, treatment, and outcomes of Aeromonas infections in Saudi Arabia.

**Methods:**

A retrospective study was performed at a tertiary university hospital with 1000 beds in Jeddah, Saudi Arabia. All patients 14 years and older with Aeromonas-positive cultures between January 1, 2015, and December 31, 2022 were included. Patient information was extracted from the electronic health records, including patient demographics, comorbidities, presenting symptoms, source of infection, human immunodeficiency virus status, culture results and antimicrobial susceptibility, use of immunosuppressive medication, and 30-day mortality.

**Results:**

In total 24 patients were identified with *Aeromonas hydrophila*-positive cultures, 22 of which were males (91.7%), and most (75%) had hospital-acquired infections. The 30-day mortality was 20.8%. All Aeromonas cultures were susceptible to gentamicin, cefepime, and ciprofloxacin, while the majority were resistant to ceftazidime (83.3%) and meropenem (62.5%). The most common disease presentation was skin and soft tissue infection (33.3%), the most common clinical sign was fever (58.3%), and the most common symptom was abdominal pain (37.5%). Comorbidities were very common (median 3, range 1–7). Pitt bacteremia score (*p* < 0.001), Charlson weighted comorbidity index (*p* < 0.02), international normalized ratio (*p* < 0.005), and the number of comorbidity factors (*p* < 0.05) were all associated with 30-day mortality due to Aeromonas infection. The number of comorbidities had the best predictive value (83.3%) of 30-day mortality (*p* < 0.05, Odds ratio 3.253, 95% confidence interval: 1.088–9.729).

**Conclusions:**

*Aeromonas hydrophila* is an important pathogen to consider in nosocomial infections. The number of comorbidities had the best predictive value of 30-day mortality. The susceptibility pattern of this organism indicates that, in Saudi Arabia, when an Aeromonas infection is suspected, treatment with quinolone along with other broad-spectrum antibiotics should be started until the culture and susceptibility results are known.

**Supplementary Information:**

The online version contains supplementary material available at 10.1186/s12879-023-08660-8.

## Background

*Aeromonas hydrophila* is a facultative anaerobic, oxidase-positive, gram-negative bacterium that is commonly isolated from fresh and brackish water. It has also been isolated from hospital water supplies and chlorinated tap water [1–3]. *Aeromonas hydrophila, A. caviae*, and *A. veronni* are part of the mesophilic Aeromonas species that grow at 35–37 °C and are the three most common Aeromonas species associated with human infections, while psychrophilic Aeromonas species grow at 22–25 °C and are associated with fish infections [1].

*Aeromonas* infection can cause a wide range of diseases, including gastroenteritis, blood-borne infections, skin and soft tissue infection, pneumonia, and peritonitis. The majority of the infections are found in patients with underlying diseases such as a malignancy or liver disease, or that are immunocompromised, but Aeromonas infections have also been reported in healthy individuals [1, 2, 4–8]. Aeromonas infections are treated with antibiotics, and although the organism is usually susceptible to several different classes of antibiotics, it can also harbour β-lactamases resulting in carbapenem resistance [9, 10].

Mortality due to Aeromonas septicemia is reported to be 32 to 45% in immunocompromised patients, such as patients with malignancies or that are neutropenic. Those patients often had skin and soft tissue infection, gastrointestinal infection, or infection caused by indwelling devices. In patients with a history of trauma or burns in combination with multiple comorbidities the mortality is reported to be around 60%. Mortality in patients with Aeromonas septicemia that are otherwise healthy are reported to be below 20% [1]. Globally, the data published on Aeromonas infections are limited, and no studies have been published about the local situation in Saudi Arabia.

The aim of this study was to investigate the risk factors, clinical presentation, treatment, and outcomes of *Aeromonas hydrophila* infections in Saudi Arabia to increase awareness of its clinical importance.

## Methods

### Data collection

This retrospective study took place at King Abdulaziz University Hospital, a hospital with 1000 beds located in Jeddah, Saudi Arabia. All patients 14 years and older with Aeromonas-positive cultures from any site were included between January 1, 2015, and December 31, 2022. The microbiology lab at King Abdulaziz University Hospital identified the clinical samples that were Aeromonas-positive. Patient information was extracted from the electronic health records, including patient demographics, comorbidities, presenting symptoms, source of infection, human immunodeficiency virus (HIV) status, the reason for admission, culture results including antimicrobial susceptibility, concomitant bacterial or fungal infection, the antibiotic used for treatment, use of immunosuppressive medication, and 30-day mortality. The route of acquiring the infection was also defined: community-acquired, if the infection developed without a record of hospitalization within the 90 days prior to presentation, or hospital-acquired, if the infection developed 48 h or more following admission or within 90 days after discharge. All procedures were carried out in accordance with relevant guidelines and regulations of the institute and the declaration of Helsinki. The study was approved (reference number: 303 − 23) by the hospital’s ethical review committee (Unit of Biomedical Ethics, Research Ethics Committee) of King Abdulaziz University in Jeddah, Saudi Arabia. The need for informed consent was waived by the hospital’s ethical review committee (Unit of Biomedical Ethics, Research Ethics Committee) of King Abdulaziz University, because of the retrospective nature of the study.

### Identification of the isolates and antibiotic susceptibility testing

*Aeromonas* strains were grown for 24 h on blood & MacConky agar plates and then identified with reference to the Clinical and Laboratory Standards Institute (CLSI) Criteria [11, 12]. VITEK 2 compact (bioMérieux, France) identified the isolates, and MALDI-TOF MS (bioMérieux, France) was used for further confirmation. VITEK 2 Compact AST GN67 and XN04 test kits (bioMérieux, France) were used to conduct antimicrobial susceptibility tests with an automated system. The Minimal Inhibitory Concentration (MIC) was measured using the CLSI Criteria [11, 12]. The antimicrobial stewardship program at King Abdulaziz University Hospital only required 8 of the 15 antibiotics from the CLSI criteria to be tested. All strains were analysed at the time of isolation; no strains were preserved for later analysis.

### Statistical analysis

Categorical data were presented as frequencies and percentages. Numerical data were presented as the mean and standard deviation. Numerical variables were checked for normality using the Kolmogorov-Smirnov test, Shapiro-Wilk test, histogram, skewness, and M outlier plots. The variables were found to be fairly normally distributed. A Student t-test compared alive and deceased patients in terms of numerical variables. The number of comorbidity factors was calculated by assigning 1 point to each comorbidity factor and adding them up. The association between infection type and mortality was assessed by Fisher’s exact test. The same test was used to assess the association between the way of infection acquisition and mortality. A Mann-Whitney U test assessed the association between infection type, way of infection acquisition and Charlson comorbidity index. A binomial logistic regression test identified the factors responsible for predicting mortality. The analyses were done with 95% confidence intervals and using IBM SPSS version 24.0. A p-value < 0.05 was considered statistically significant.

## Results

### Patients and infections

**Fig. 1:**
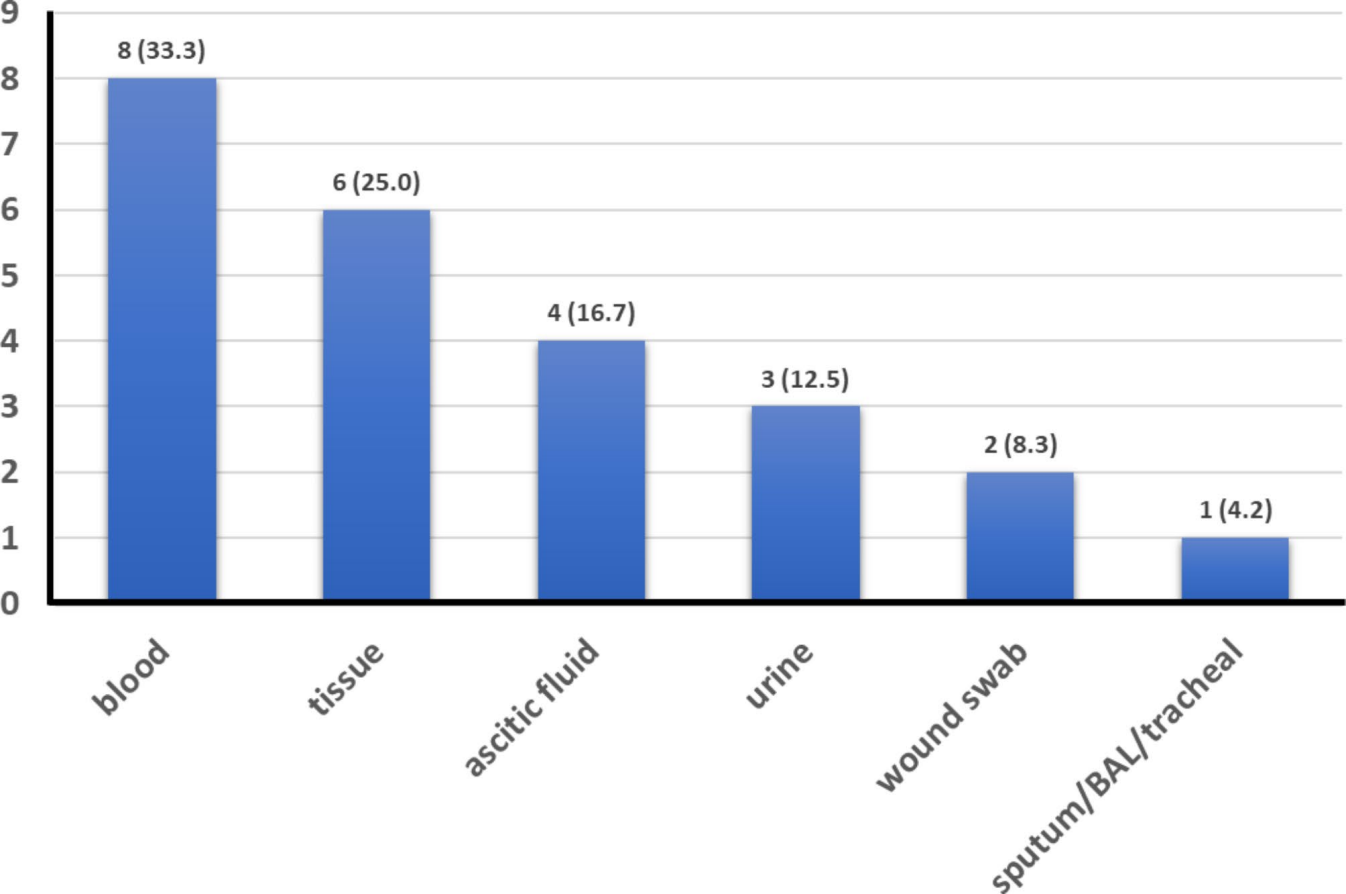
Distribution of tissues from which the *A. hydrophila* strains were isolated, n (%) Data of the 24 *A. hydrophila* strains isolated. The y-axis indicates the number of cases identified in each specific tissue. BAL, bronchoalveolar lavage

This retrospective study identified 24 cases of *Aeromonas hydrophilia* infection. No infections with other Aeromonas species were identified. Most of the cases were male (n = 22, 91.7%), had hospital-acquired, nosocomial infections (n = 18, 75%), and had polymicrobial cultures (n = 13, 54.2%) (Table 1). The cases were admitted to the surgery ward (25%), emergency room (37.5%), medical ward (12.5%), and other wards (25%). Of the culture sites, the most common site was blood (33.3%), and the least common site was sputum/bronchoalveolar lavage/tracheal (4.2%) (Fig. 1). Five patients died within 30 days, so the 30-day mortality was 20.8%. Samples from three of the five deceased patients had polymicrobial cultures, the other two had monomicrobial cultures.

**Table 1:** Distribution of categorical variables

Categories	Attributes	N	%
Sex	Male Female	22 2	91.7 8.3
Infection acquired	Community-acquired Hospital-acquired	6 18	25.0 75.0
Type of infection	Monomicrobial Polymicrobial	11 13	45.8 54.2

**Fig. 2:**
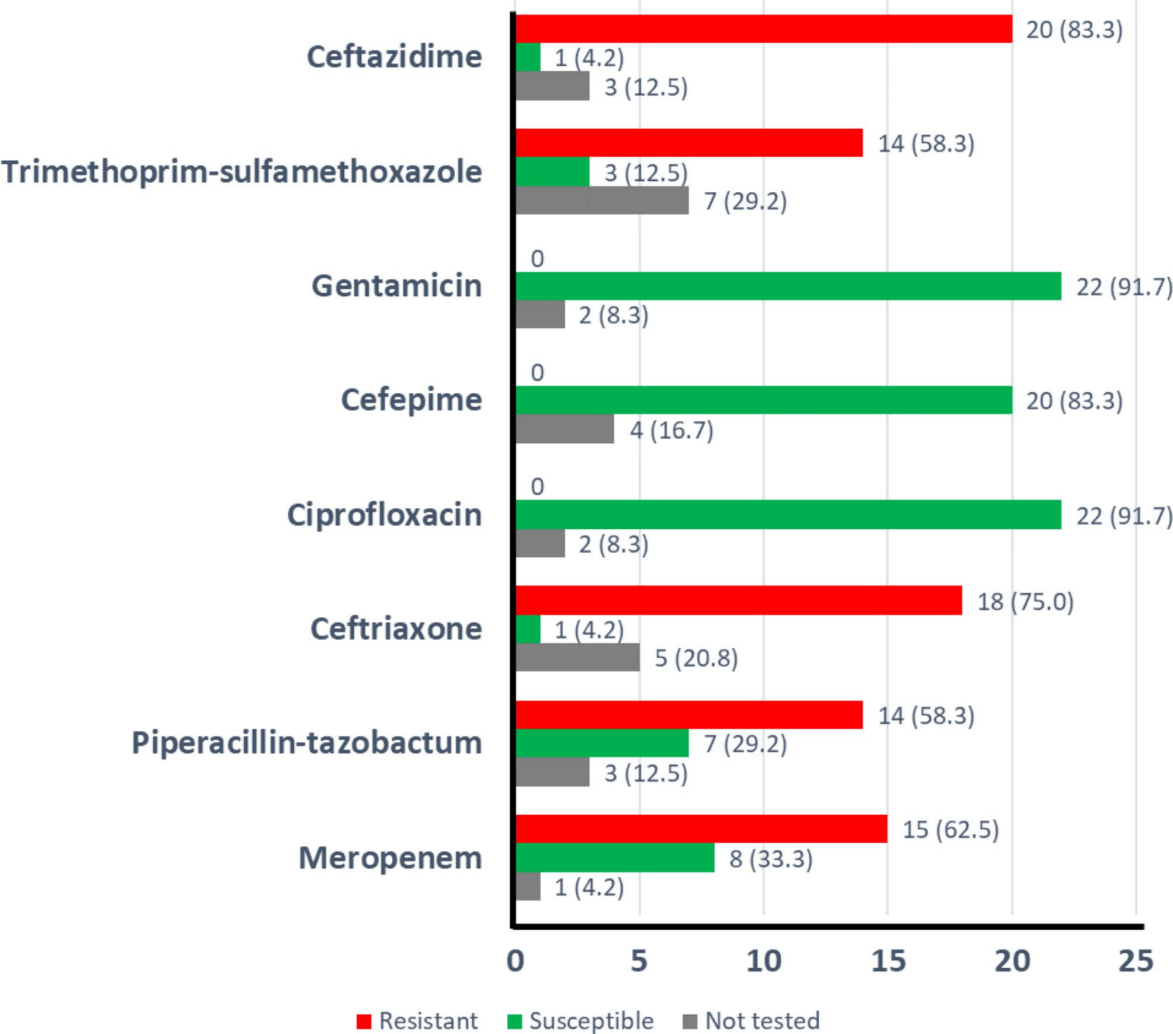
Susceptibility to antibiotics of the 24 *A. hydrophila* strains, n (%) The x-axis indicates the number of strains resistant, susceptible or not tested for each antibiotic

Antibiotic susceptibility testing showed 0% resistance of the *A. hydrophila* strains to gentamicin, cefepime, and ciprofloxacin (Fig. 2). In contrast, 83.3% of specimens were resistant to ceftazidime, 75% to ceftriaxone, and 62.5% of strains were resistant to meropenem (Fig. 2).

### Disease presentation, clinical signs and symptoms, and comorbidities

**Fig. 3:**
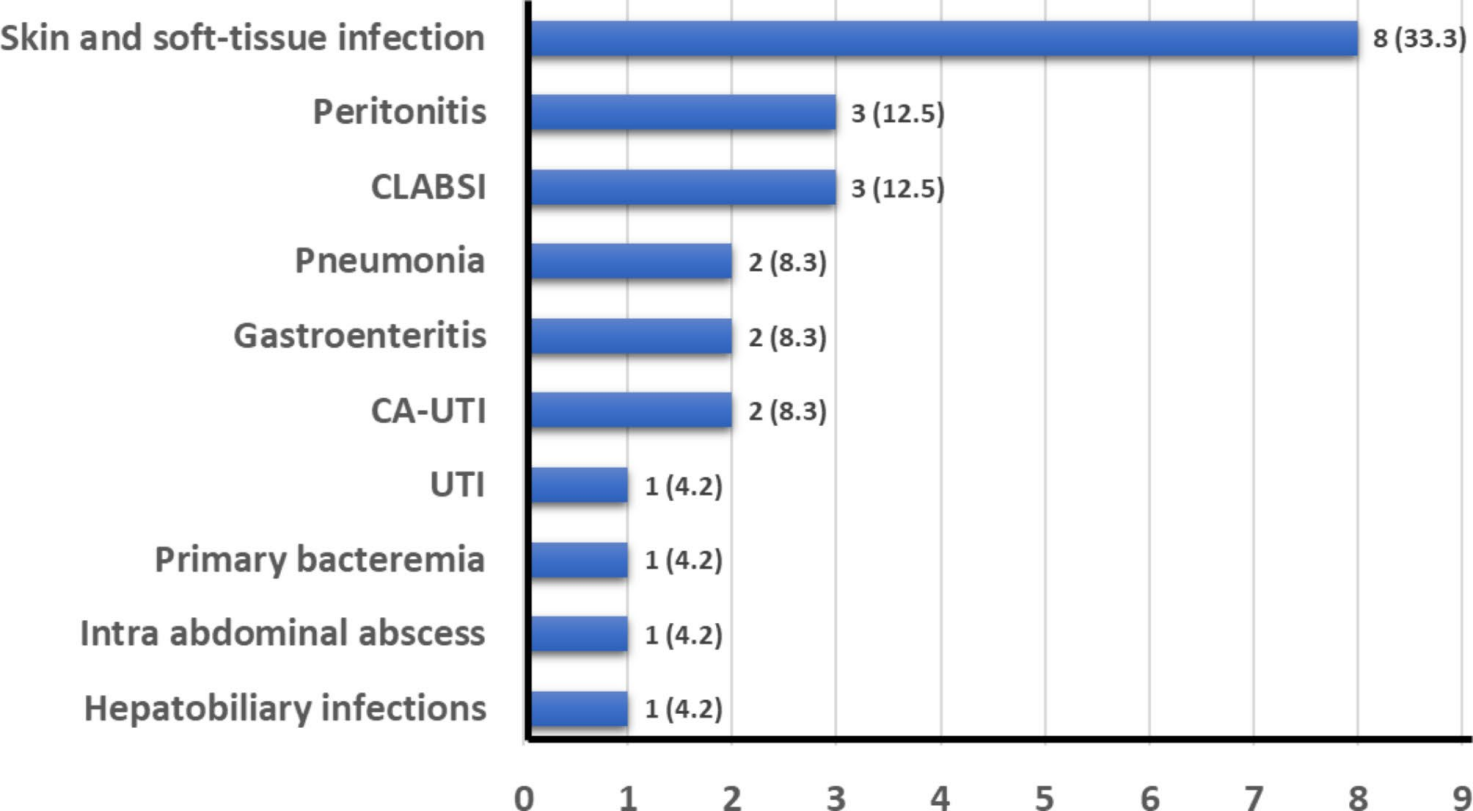
Disease presentation of all cases, n (%) The x-axis indicates the number of cases in which each disease presentation was observed. CA-UTI, catheter associated-urinary tract infection; CLABSI, central line-associated bloodstream infection; UTI, urinary tract infection

The most common disease presentations were skin and soft tissue infection in eight cases (33.3%), followed by peritonitis in three cases (12.5%), and central line-associated bloodstream infection (CLABSI) in three cases (12.5%). All other presentations occurred only once or twice (Fig. 3).

**Fig. 4:**
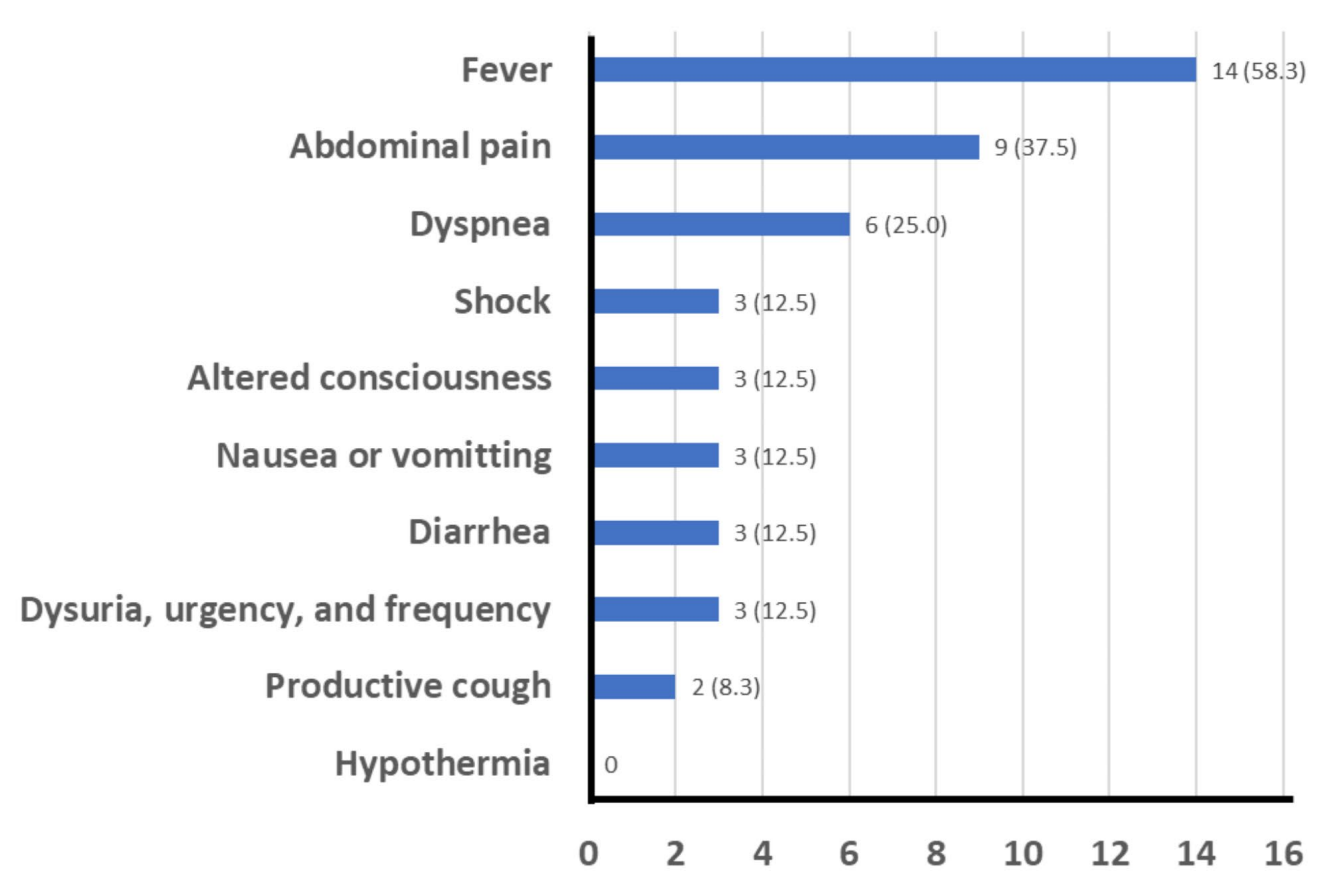
Clinical signs and symptoms observed in the patients, n (%) The x-axis indicates the number of reported signs or symptoms

The most prevalent clinical sign Aeromonas septicemia was fever in 14 cases (58.3%), and the most prevalent symptoms were abdominal pain in nine cases (37.5%), and dyspnea in six cases (25%) (Fig. 4).

**Fig. 5:**
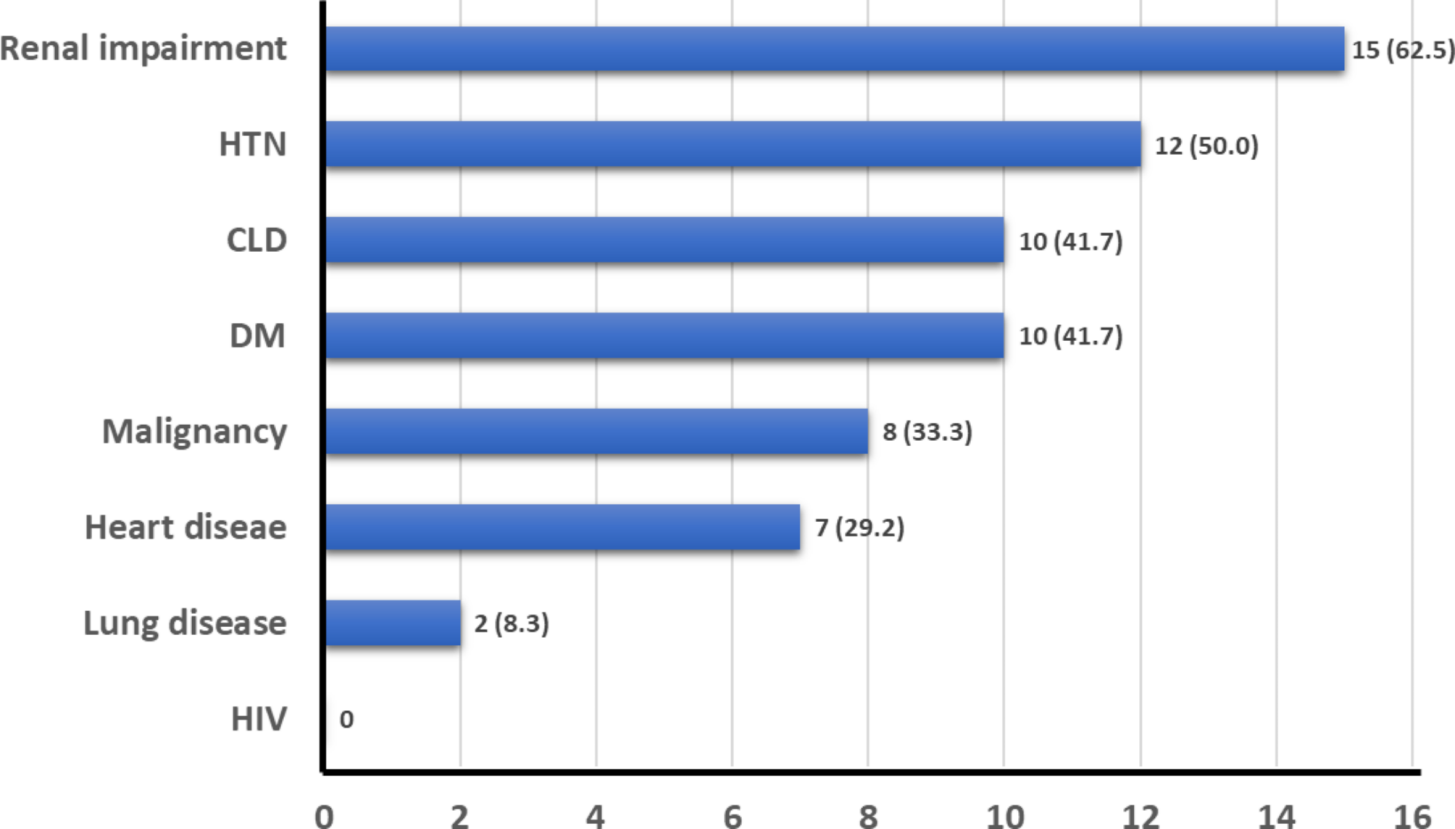
Comorbidities observed in all patients, n (%) Note: patients can have more than one comorbidity. CLD, chronic liver disease; DM, diabetes mellitus; HIV, human immunodeficiency virus; HTN, hypertension

Comorbidities were common; each patient had at least one comorbidity (median 3, range 1–7). Almost two-thirds of the cases (62.5%) had a renal impairment (five of which were on hemodialysis), and half (50.0%) had hypertension (Fig. 5).

There was no significant association between type of infection (monomicrobial and polymicrobial) and mortality, or between infection acquisition (hospital-acquired or community-acquired) and mortality (Table S1).

### Several continuous variables were associated with mortality

Continuous variables for all cases and for the two 30-day mortality outcomes (dead, alive) are presented in Table 2. The mean age of all cases was 49.79 ± 21.89 years, the mean Charlson weighted comorbidity index was 4.38 ± 2.90, and the mean Pitt bacteremia score was 4.33 ± 4.85.

Between the deceased and alive cases, there were several statistically significant differences: the Pitt bacteremia score (*p* < 0.001, 95% CI = -13.41–7.59), Charlson weighted comorbidity index (*p* = 0.019, 95% CI = -6.04–0.59), international normalized ratio (INR) for blood clotting (*p* = 0.004, 95% CI = -3.33–0.71), and the number of comorbidity factors (*p* = 0.020, 95% CI = -3.82–0.36). All these scores were significantly higher among deceased cases compared to the alive cases (Table 2).

Two of the eight patients with bacteremia (25.0%) and three of the sixteen patients without bacteremia (18.8%) died. This difference in mortality was not statistically significant (Chi-square test, *p* = 0.725).

**Table 2:** Distribution of continuous variables in all cases and based on mortality outcome

	All cases		Mortality = yes		Mortality = no				
Variables	Mean	SD	Mean	SD	Mean	SD	p-value*	Mean difference	95% CI
Age, in years	49.79	21.89	52.00	24.48	49.21	21.84	0.806	-2.79	-26.08–20.50
Pitt bacteremia score	4.33	4.85	12.50	2.12	2.00	1.41	< 0.001	-10.50	-13.41–7.59
Charlson weighted comorbidity index	4.38	2.90	7.00	0.00	3.68	2.89	0.019	-3.32	-6.04–0.59
WBC, in k/μL	28.92	58.33	55.83	99.26	21.84	43.63	0.255	-33.98	-94.31–26.34
Platelet count, in k/μL	250.75	233.02	171.00	197.50	271.74	241.83	0.402	100.74	-143.59–345.07
AST, in u/L	73.75	105.28	137.20	146.61	57.05	89.30	0.133	-80.15	-186.61–26.32
Bilirubin, in μmol/L	28.58	31.16	47.00	35.80	23.74	28.93	0.141	-23.26	-54.84–8.32
INR	1.62	1.48	3.22	2.52	1.20	0.71	0.004	-2.02	-3.33–0.71
Creatinine, in μmol/L	294.29	338.80	422.60	291.24	260.53	349.35	0.353	-162.07	-515.99–191.84
Duration of antibiotic treatment, in days	7.58	5.17	8.00	6.63	7.47	4.94	0.845	− 0.53	-6.04–4.98
Number of comorbidities	3.54	1.84	5.20	1.30	3.11	1.73	0.020	-2.09	-3.82–0.36

AST, aspartate aminotransferase; INR, international normalized ratio; SD, standard deviation; WBC, white blood cells

*based on independent samples t-tests

### The number of comorbidity factors predicted mortality

A binomial logistic regression analysis was done to identify factors predictive of mortality. Due to the small number of cases only a limited number of variables could be included in the analysis. Age, type of infection (monomicrobial or polymicrobial), and the number of comorbidity factors were included in the analysis as these are in general considered to be related to mortality outcome. The overall model prediction was 83.3%. The analysis showed that the number of comorbidity factors was predictive of mortality, at *p* = 0.035, odds ratio = 3.253, 95% CI = 1.088–9.729 (Table 3).

**Table 3:** Binary logistic regression of potential risk factors

Factor	*p*-value	Odds ratio	95% CI	
			Lower limit	Upper limit
**Age**	0.350	0.970	0.911	1.033
**Type of infection** Monomicrobial vs. polymicrobial	0.307	4.527	0.249	82.266
**Number of comorbidities**	0.035	3.253	1.088	9.729

CI, confidence interval

## Discussion

*Aeromonas hydrophila* risk factors, clinical disease, and factors associated with mortality were studied in a large hospital in Saudi Arabia. This is the first study from this region about this particular species. The Pitt bacteremia score, Charlson weighted comorbidity index, INR, and the number of comorbidity factors were all found to be associated with 30-day mortality due to Aeromonas infection. Of these risk factors, the number of comorbidity factors had the best predictive value for 30-day mortality due to Aeromonas infection.

In general, patients accumulate more comorbidities with advanced age, thus increasing the risk of 30-day mortality. In this retrospective study, each patient had at least one comorbidity. In a prospective study of 78 individuals that tested positive for Aeromonas species in France, the majority of individuals (61.5%) did not have any comorbidities [7]. Most of the patients in the current study had renal impairment, followed by hypertension, chronic liver disease, and diabetes. These comorbidities are different from a larger study in Taiwan, where they found chronic liver disease (54%) and malignancy (22%) as the most common comorbidities associated with Aeromonas bacteremia [13] and the French study, where malignancy (19.2%) and immunosuppression (14.1%) were the most common comorbidities [7]. These differences in comorbidities may partly be explained by the fact that in the French study, only 35.7% of cultures contained *A. hydrophila* [7], and in the Taiwanese study, only 58% of cultures contained *A. hydrophila* [13], as the various Aeromonas species proved to have different disease presentations [7]. *A. hydrophilia* was mostly (76%, 19/25) found in wound and skin soft tissue infections, while for instance *A. veronii* and *A. caviae* were found mostly in other sites and only in 43% (12/28) and 20% (3/15) respectively in wound and skin soft tissue infections. These differences suggest the disease presentations, including the comorbidities, cannot easily be compared between the patients from the different studies as they did not all have the same Aeromonas infections. In addition, there may be genetic and cultural differences between the patients in these countries that explain these differences.

In terms of the clinical picture, the most common sign encountered in the current study was fever secondary to central line infection, and the most common symptom was abdominal pain, followed by dyspnea. Only 12% of the patients had diarrhoea, in contrast to studies where diarrhoea was usually named as the most common symptom (although percentages are not given) in adults and children [1, 8]. This diarrhoea is self-limiting and sometimes associated with chronic colitis [1, 14]. In Spain, Aeromonas was found to be the fourth most common cause of gastroenteritis among microbiological causes of gastroenteritis (although that did not tell us how many of the Aeromonas cases had gastroenteritis) [15]. In the French study, only 19% of patients had gastroenteritis [7], while in an earlier Taiwanese study only 5% had diarrhoea as presenting symptom [16]. Together these results suggest that diarrhoea is not as common in Aeromonas infection as previously thought. Further studies are needed in larger numbers of patients to determine whether this is indeed the case.

In the patients, 75% of the invasive infections associated with wounds were hospital-acquired, nosocomial infections rather than community-acquired infections. In contrast, in a study of hospitalised patients in India, only 19% of the infections were hospital-acquired [17]. In a study in France about half of the wound and soft tissue infections were due to freshwater exposure [7]. It can be speculated that in Saudi Arabia the lack of fresh open water (such as lakes and rivers) reduces the chances of developing an environment-acquired *A. hydrophilia* infection.

The 30-day mortality was, at 20.8%, not high in the patients in the current study compared to other studies, although not easy to compare as different time frames were used: For instance, in the study in Taiwan, 14-day mortality was 32% [13], and in a study in Spain 1-year mortality was 26.5% [18]. Overall, the reported mortalities ranged between 25% and 46% in cases with bacteremia and were about 50% in cases with pneumonia [2, 4, 16, 19–22]. The low mortality rate in the current study can be attributed to the fact that most of the cases had skin and soft tissue infections, which has a relatively good prognosis, while none of them had necrotizing fasciitis, and few cases had pneumonia (12%), which are usually associated with high mortality.

The factors associated with 30-day mortality in this study were high Pitt bacteremia scores, a high Charlson weighted comorbidity index, high INR, and a large number of comorbidity factors. In the study in Spain, age, in-hospital patient, ICU stay, extraintestinal presentation, malignancy, and antimicrobial treatment were associated with increased mortality [18]. In the study in Taiwan, the strongest association was found with initial serum creatinine, the number of positive blood cultures, and the severity score [13]. Comparisons between the factors associated with mortality were hampered by a difference in percentage of patients with *A. hydrophila*, as this was 100% in the current study, 58% in the Taiwanese study, and unknown in the Spanish study. In addition, it was previously found that the various Aeromonas species have different clinical presentations [7], which may also affect factors associated with mortality.

Several studies reported that most patients developed Aeromonas infection following trauma or environmental exposures, as Aeromonas is found in the environment and soil [23–25]. In the current study, none of the patients with skin and soft tissue infections had a history of trauma, and their infections were considered secondary to underlying comorbidities. A similar result was also reported in a study in which less than 3% of patients had skin and soft tissue infections due to surgical or traumatic wound infection [18].

Antibiotic susceptibility testing showed 0% resistance to gentamicin, cefepime, and ciprofloxacin in this study. In contrast, 83.3% of specimens were resistant to ceftazidime, 75% to ceftriaxone, and 62.5% to meropenem. In an extensive review, Aeromonas was reported to be uniformly resistant to ampicillin, penicillin, and cefazolin, and was reported to have variable susceptibility to piperacillin-tazobactam as Aeromonas can produce β-lactamases including Ambler class D penicillinases, class C cephalosporinases, and TEM family extended spectrum β-lactamases [1]. We found zero resistance to gentamicin, which is in line with the observation that aminoglycosides (e.g., gentamicin) are usually active agents against Aeromonas [26]. Quinolones (e.g., ciprofloxacin) were also reported to work very well for Aeromonas species as resistance against quinolones is uncommon [27], which we confirmed in this study (0% resistance). Resistance to meropenem was low at 7% in Korea [20], whereas it was much higher in this study (62.5%). In the same study, resistance to ceftriaxone and piperacillin-tazobactam was 15.5% which was very different from what we found in the current study as both ceftriaxone resistance and piperacillin-tazobactam resistance were high at respectively 75% and 58.3% [20]. In Spain, similar results of high susceptibility to both cefepime and gentamicin were found [18], matching the results in the current study. Apparently, the resistance pattern of Aeromonas in Saudi Arabia is different from other regions. We speculate that the resistance patterns may be different between the regions due to a difference in antibiotic prescription and antimicrobial stewardship, causing the resistance to rise in some regions in comparison to others. Comparing genomic sequences of the strains from different countries may answer this question. When Aeromonas infection is suspected, treatment with a quinolone along with another broad-spectrum antibiotic, such as piperacillin/tazobactam or meropenem, can be started until the culture and susceptibility results are known.

The current study had several limitations. Firstly, the small number of patients. As Aeromonas infection is very rare, and only cases were included that had invasive disease while any Aeromonas-positive cases that appeared to be due to colonization rather than a true infection were excluded, a small number of patients was analysed. This made it difficult to do meaningful statistical analyses. Secondly, no genomic testing was performed to assess the molecular basis of resistance. Genomic testing may have given a better picture of common resistant mechanisms in Saudi Arabia. And thirdly, the study was performed in a single hospital, so the results cannot be generalised to other hospitals. A future large, prospective, multi-center study could identify larger numbers of patients, thus resulting in more robust data.

A strength of the study was that it was the first study of its kind in Saudi Arabia, and performed at a large University teaching hospital. So far, very little was known about Aeromonas infections and their clinical implications in Saudi Arabia.

## Conclusions

This study highlighted that *Aeromonas hydrophila* is an important pathogen to consider in nosocomial infections and that the risk of 30-day mortality increased with the number of comorbidities, Charlson weighted comorbidity index, and Pitt bacteremia score, of which the number of comorbidities had the best predictive value. The results showed that, in Saudi Arabia, the susceptibility pattern of this organism indicates that if Aeromonas infection is suspected, definitely a quinolone should be added to the regimen along with other broad-spectrum antibiotics until the culture and susceptibility results are known.

### Electronic supplementary material

Below is the link to the electronic supplementary material.

Supplementary Material 1

## Data Availability

The datasets used and analysed during the current study are available from the corresponding author on reasonable request.

## Abbreviations

CLABSI: Central line associated bloodstream infection
CLD: Chronic liver disease
CLSI: Clinical and Laboratory Standards Institute
DM: Diabetes mellitus
HIV: Human immunodeficiency virus
HTN: Hypertension
INR: International normalized ratio
MIC: Minimal inhibitory concentration
SD: Standard deviation
UTI: Urinary tract infection
WBC: White blood cells
