# Supplementary material for: A retrospective study of Aeromonas hydrophila infections at a university tertiary hospital in Saudi Arabia
Source: BMC Infect Dis. 2023 Oct 9;23:671. doi: 10.1186/s12879-023-08660-8 (PMC10563259; doi:10.1186/s12879-023-08660-8)
Supplement: Supplementary file 1 — Supplementary Material 1 [file 12879_2023_8660_MOESM1_ESM.docx]

**Supplemental material**

## **Table S1 Distribution of categorical variables in all cases and based on mortality outcome**

|  |  | **All cases** | |  | **Mortality: yes** | |  | **Mortality: no** | |  |  |
| --- | --- | --- | --- | --- | --- | --- | --- | --- | --- | --- | --- |
| **Categories** | **Attributes** | **N** | **%** |  | **n** | **%** |  | **n** | **%** | ***p*-value** | **Odds ratio** |
| Infection acquired | Community-acquired  Hospital-acquired | 6  18 | 25.0  75.0 |  | 5  0 | 27.8  0.0 |  | 13  6 | 72.2  100.0 | 0.280 | 1.385 |
| Type of infection | Monomicrobial  Polymicrobial | 11  13 | 45.8  54.2 |  | 2  3 | 18.23.1 |  | 9  10 | 81.8  76.9 | 0.999 | 1.350 |

N, number
